# Genome Survey Sequencing of the Mole Cricket *Gryllotalpa orientalis*

**DOI:** 10.3390/genes14020255

**Published:** 2023-01-18

**Authors:** Kuo Sun, De-Long Guan, Hua-Teng Huang, Sheng-Quan Xu

**Affiliations:** 1 College of Life Sciences, Shaanxi Normal University, Xi’an 710062, China; 2 School of Chemistry and Bioengineering, Hechi University, Hechi 546300, China

**Keywords:** genome size, flow cytometry, k-mer, genome survey

## Abstract

The mole cricket *Gryllotalpa orientalis* is an evolutionarily, medicinal, and agriculturally significant insect that inhabits underground environments and is distributed globally. This study measured genome size by flow cytometry and k-mer based on low-coverage sequencing, and nuclear repetitive elements were also identified. The haploid genome size estimate is 3.14 Gb by flow cytometry, 3.17 Gb, and 3.77 Gb-based two k-mer methods, respectively, which is well within the range previously reported for other species of the suborder Ensifera. 56% of repetitive elements were found in *G. orientalis*, similar to 56.83% in *Locusta migratoria*. However, the great size of repetitive sequences could not be annotated to specific repeat element families. For the repetitive elements that were annotated, Class I-LINE retrotransposon elements were the most common families and more abundant than satellite and Class I-LTR. These results based on the newly developed genome survey could be used in the taxonomic study and whole genome sequencing to improve the understanding of the biology of *G. orientalis*.

## 1. Introduction

A total of 29,000 species comprises the Orthoptera, which is divided into two suborders: Ensifera (crickets, katydids, and weta) and Caelifera (grasshoppers and locusts). Mole crickets (Ensifera: Gryllotalpidae) are a small monophyletic group within the Gryllidea clade [1] that consists of more than 100 species in six genera across the globe [2]. Gryllotalpids are pest insects adapted to living underground and characterized by unique morphological characteristics (digging forelegs [3], tumescent pronotum, short antennae, and hind legs that are incapable of jumping [4]).

**Figure 1:**
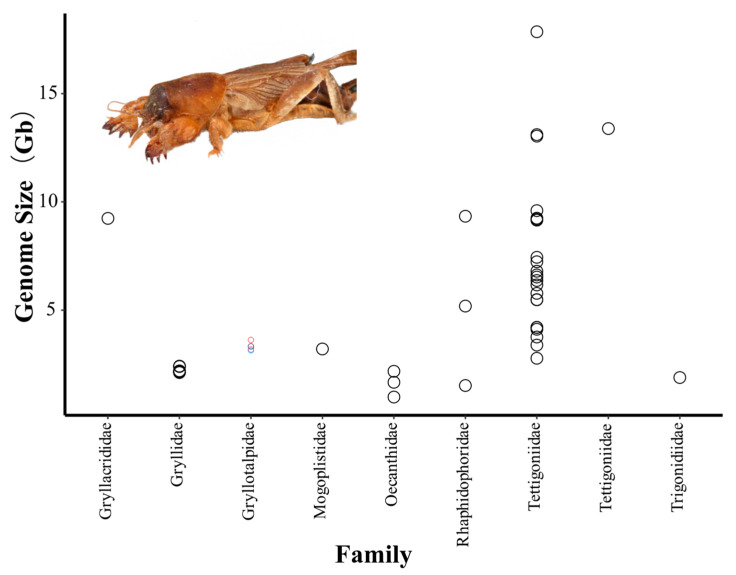
Genome size (GS) estimation using flow cytometry (blue circle) and k-mer (red circle, GS estimated by KmerGenie; purple circle, GS estimated by GenomeScope) approach of the mole cricket, *G. orientalis*, and GS estimates of other species belonging to different families in the suborder Ensifera (black circles). GS of other Ensifera obtained from a previous study [13] and Animal Genome Size Database (www.genomesize.com) accessed on 14 November 2022. A specimen of *G. orientalis* is shown in the inset at the top.

*G. orientalis*, a species of mole cricket that inhabits predominantly Asian countries but also lives in European and African countries [5], causes damage to crops such as potatoes, sugar canes, and Chinese yam [6,7] (Figure 1). Previously, detailed studies of their life history have already been extensively investigated [8,9,10]. A seasonal wing polymorphism control mechanism was described for them. From mid-June to September, the long-winged morph emerges, while from September to mid-June, the short-winged morph appears [11]. In addition, there have been investigations of the burrows of *G. orientalis* (shallow horizontal and deep vertical burrows) under different environmental conditions and during various seasons to determine their functions [12]. For instance, mole crickets often modify or change their burrow structures or burrowing sites to meet their needs, such as shallow horizontal and deep vertical burrows being used for foraging and horizontal burrows being used for escape and mating. The *Gryllotalpa* species were previously reported as one of the most distinctive Orthoptera species [1]. Thus, newly added insect genomic surveys are expected to provide further insight into insect genome diversity and evolution.

This study is part of a broad effort to develop genomic resources in mole crickets. We present the first *G. orientalis* genome survey using a low-coverage shot-read next-generation sequencing approach. We estimated its genome size (GS) using two approaches based on k-mers and flow cytometry (FCM) and made a detailed comparison of GS variation among Ensifera families. In this study, nuclear repetitive elements were identified, annotated, and characterized in this species for the first time. Additionally, microsatellites or short sequence repeats (SSRs) were identified. Genome survey resources are essential for improving the understanding of biology in mole crickets and also can contribute to future whole genome sequencing of this species.

## 2. Materials and Methods

### 2.1. Specimen Collection and DNA Extraction

Samples of *G. orientalis* used in this study were collected from natural populations (Hubei, China, 110.79° N, 32.65° W) and stored in liquid nitrogen in the Insect Laboratory of Shaanxi Normal University. In accordance with the manufacturer’s instructions, DNeasy Blood and Tissue Kit (Qiagen, Germantown, MD, USA) was used to extract genomic DNA from muscle tissue.

### 2.2. Library Preparation and Sequencing

The standard protocol of the NEBNext Ultra DNA Library Prep Kit for Illumina (New England BioLabs, Hitchin, UK) was used to prepare the Illumina paired-end (PE) shotgun DNA library and then sequenced on the Illumina HiSeq X Ten platform (Illumina, San Diego, CA, USA) with a 150 bp read length. Reads after filtering are available in the National Genomics Data Center repository (Bioproject ID: PRJCA013546; BioSample accession: SAMC1008585).

### 2.3. Measuring Genome Size Using Flow Cytometry (FCM)

We followed the FCM protocol described by Gregory and Hare [14,15]. Because the survey results showed that mole crickets have large GS (see results), we used the migratory locust (*L. migratoria*, 1C = 6.5 Gb) as internal standards [16], and male adult *G. orientalis* samples were used for GS estimates. To ensure the accuracy of the measurement, we first measured the samples containing only the head tissue of locust or mole cricket separately to check that the fluorescence intensity of the 4C peak was twice that of the 2C peak. Firstly, head tissue was placed in a 1 mL cold Galbraith buffer [17] and ground with a glass tissue grinder (20 strokes). Then, we filtered the grounded solution through a 38 mm nylon mesh and stained it for at least 30 min with 0.01 mg/mL propidium iodide on ice in the dark. The fluorescence intensity of samples was measured on the CytoFLEX flow cytometer (Beckman-Coulter) using only red fluorescent images (488 nm). To measure the GS of *G. orientalis*, heads of internal standard and *G. orientalis* were also placed in a 1 mL cold buffer. The following steps are consistent as previously described.

### 2.4. Estimating Genome Size Based on k-mer in G. orientalis

Using the default parameters of fastp v.0.20.1 [18], contaminants, low-quality sequences, and adapters were filtered. We use FastQC [19] to evaluate the quality of the reads after trimming. A total of 377,197,730 high-quality read pairs (113.16 Gb, ~36×) were obtained (Q30 > 90%; GC content 40%). We used these clean PE reads to estimate GS using KmerGenie [20] and GenomeScope [21], respectively.

### 2.5. Repetitive Elements in G. orientalis

As described in Baeza [22], the repetitive elements of *G. orientalis* were identified, annotated, and quantified using RepeatExplorer [23,24] to cluster similar reads on Galaxy platform, http://repeatexplorer.org/ (accessed on 22 November 2022). The RepeatExplorer program provided a rapid analysis of plant and animal genomes’ repeat composition and abundances. Clusters were annotated by RepeatMasker (http://repeatmasker.org) using the Metazoa version 3.0 database [25]. Reads within each cluster are assembled by CAP3 (options: -O -p 80 -o 40) [26]. RepeatExplorer was configured with default values for all other parameters. We first pre-ran 20,000 sample reads to estimate the maximum number of reads that can be analyzed and then sample 2,655,436 reads with 64 RAM to estimate the proportion of each repetitive element in *G. orientalis*.

### 2.6. Discovering Microsatellite in G. orientalis

The pipeline Pal_finder [27] was applied in the Galaxy platform (https://palfinder.ls.manchester.ac.uk, accessed on 18 November 2022) to identify simple sequence repeats (SSRs) in mole cricket’s genome. Sequences containing repeat motifs are identified using Pal_finder version 0.02.04 [27]. Optimal SSR loci were selected using the pal_filter with default settings and the strictest filtering parameters that only include loci with designed primers. Remove loci where primer sequences occur multiple times in the reads, and only include loci with ‘perfect’ motifs—then use Primer3 [28] to develop PCR priming sites. The minimum number was set to 5 to detect SSRs of 2-mer repeat units and a minimum of 6 to detect repeats of 3, 4, 5, and 6-mer repeat units. Finally, we assembled paired-end reads using PANDAseq [29] and confirmed primer sequences were present.

## 3. Results and Discussion

### 3.1. Genome Size Measuring Using FCM in G. orientalis

**Figure 2:**
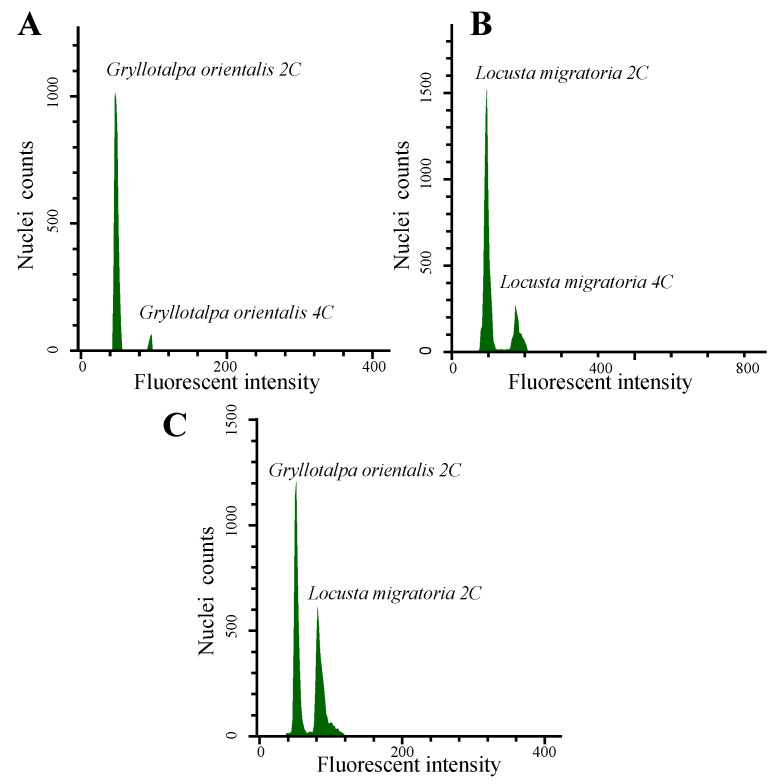
Measuring the nuclear DNA content of *G. orientalis* with *L. migratoria* as an internal standard. Flow cytometric measurement of the nuclear DNA content of *G. orientalis* with *L. migratoria* as internal standard (6.5 Gb). These histograms showed the fluorescence intensity of (**A**) 2C and 4C peaks in *G. orientalis.* (**B**) 2C and 4C peaks in *L. migratoria.* (**C**) 2C peaks in *G. orientalis* and *L. migratoria.* Nuclei’s fluorescence intensity is shown on the *x*-axis, and the number of nuclear is shown on the *y*-axis.

In the previous literature, GS in Ensifera was reported to range from 0.952 pg (or 0.93 Gb; male) to 19.135 pg (or 18.71 Gb; female) by FCM [13]. Here, we investigated the average GS of 41 Ensifera species, including nine species from the Animal Genome Size Database [30] and 32 species from a previous study [13] (Figure 1), and unified the unit of GS as Gb (1 pg = 978 M) [31]. In the suborder Ensifera, GS varies greatly from 0.99 Gb of *Oecanthus sinensis* (fam. Oecanthidae) to 17.26 Gb of *Deracantha onos* (fam. Deracantha) and shows a 17.37-fold variation. In the family Gryllotalpidae, GS varies moderately from Gb to 3.47 Gb and averages 3.31 Gb. According to protocols described by Hare [15], fluorescence measurements have been confirmed linearity by measuring the 4C peak twice as high as the 2C peak in *G. orientalis* and *L. migratoria,* respectively (Figure 2A,B). In this study, the average haploid GS of male *G. orientalis* estimated using FCM was 3.14 Gb (Figure 2C), similar to the value estimated based on k-mer (Figure 1). However, the GS of male *G. orientalis* estimated in our study was lower than 4.2 pg (or 4.1 Gb, female) by FCM in a previous study [13]. Such results may be caused by the different sex used in the measurement, and GS differences have also been reported between male and female grasshoppers [32].

### 3.2. Genome Size Estimation Using k-mer in G. orientalis

To further verify GS measurements of *G. orientalis* by FCM (Figure 2), we also estimated GS by k-mer based on the genome survey sequencing of *G. orientalis* by KmerGenie [20] and GenomeScope [21]. The most appropriate k-mer length was auto-selected by KmerGenie according to the k-mer abundances histogram (Table S1; Supplementary Material File S1). As a result, the predicted GS of *G. orientalis* was 3.77 Gb (Figure S1) by KmerGenie, and GS estimated by GenomeScope with default k-mer length 21 was 3.17 Gb (Figure S2). Sequenced Ensifera genomes varied from 929.2 Mb in *Acheta domesticus* (house cricket) [33] to 9 Gb in *Meconema thalassinum* (GenBank: GCA_946902985.1).

### 3.3. Repetitive Elements in the Nuclear Genome of G. orientalis

**Figure 3:**
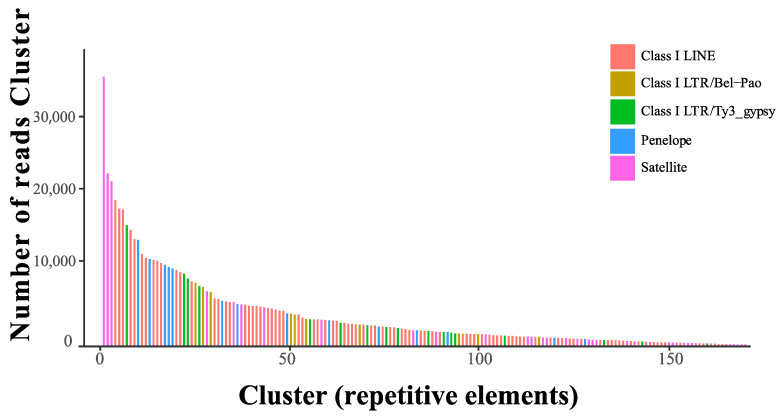
Size distribution and repeat composition of mole cricket clusters generated by similarity-based partitioning. Size distribution and repeat composition of annotated clusters generated by similarity-based partitioning in the mole crickets—*G. orientalis*. Bars are colored according to the type of repeat present in clusters based on the similarity search in RepeatExplorer2.

Two million six hundred fifty-five thousand four hundred thirty-six reads (0.25 × X) were used to analyze the repeat content of *G. orientalis* by the RepeatExplorer pipeline. The result shows that a total of 1,791,289 reads were contained in 95,375 clusters and 265 top clusters (56%, *n* = 1,487,044), representing the most abundant class of repetitive elements in the genome of *G. orientalis*. However, a lot (9.45%) of the top repetitive element families (112 top clusters, 250,946 reads) were reported as “unclassified,” which means that no repeat family could be assigned to these clusters. Mole crickets have a smaller repeat content of 56.08% compared to 74.56% in grasshopper *Angaracris rhodopa*, but similar to 56.83% in locusts while its GS was 6.5 Gb and larger than mole crickets [34]. A total of 169 clusters were annotated, and the most common repetitive elements were identified as short interspersed nuclear elements (Class I-LINE; *n* = 84 clusters, 370,704 reads), which were more common than satellite (*n* = 35 clusters; 135,440 reads), Class I-LTR-Ty3_gypsy elements (*n* = 15 cluster; 70,791 reads), Class I-LTR-Penelope (*n* = 12 cluster; 89,263 reads) and Class I-LTR-Bel-Pao (*n* = 10 cluster; 45,025 reads) (Figure 3). The TE family proportion of the total TE content in *G. orientalis* (LINE:21%; UNCLASS: 23%, LTR: 5%; SINE: 1%) was slightly lower than in Mediterranean field cricket in a recent study [35]. Annotations for DNA content were missing in *G. orientalis*, most likely due to the lack of specific custom repeat libraries for this group. This analysis revealed that *G. orientalis* still had considerably large repeat elements that were not annotated, and a custom repeat library needs to be constructed with the completion of whole genome sequencing and annotation of mole crickets in future work. *G. orientalis* had a moderately large, estimated GS relative to species observed in the Ensifera, combined with repetitive elements in high abundance. For high-quality assembly of this species’ genome, short and long reads should be considered. 

### 3.4. Microsatellite Discovery in G. orientalis

SSR is a class of genetic markers that plays an important part and has been widely used in population genetic studies [36]. A total of 2939 SSR primers pairs were identified (*N* = 2235, 501, 176, 24, 3 for 2-mer, 3-mer, 4-mer, 5-mer, and 6-mer SSRs motifs; Table S2). A study for *Gryllotalpa major* (prairie mole cricket) explored population genetics and developed 15 species-specific microsatellite DNA loci [37]. In this study, we used a more economical method rather than prior enrichment to obtain a large number of SSR loci that could be used for potential PCR-amplified, which may be helpful for the research involving population structure, genetic mapping, and evolution based on SSR in the future. SSR studies of *G. orientalis* also could combine mitochondrial protein-coding genes or whole mitochondrial genomes to assess population genomic structure throughout the distribution range of *G. orientalis* in Asia or worldwide.

## 4. Conclusions

This study developed genomic survey resources of the mole cricket for the first time. *G. orientalis* is an evolutionarily, medicinal, and agriculturally significant underground insect. Genome size was estimated using low-coverage short-read Illumina sequencing and flow cytometry. However, the GS estimation based on k-mer analysis exhibits bias, which may be caused by the limitations of next-generation sequencing data. Therefore, flow cytometry should be recommended for estimating the genome size of *G. orientalis*. Nuclear repetitive elements were identified and partially classified, and a specific repeat library for the mole cricket should be constructed in future work. Additionally, a large number of SSRs were identified, and these SSRs are useful for potentially amplifiable SSR loci. All the information above will also contribute to a chromosome-level genome assembly of *G. orientalis* and help us better understand this fossorial Ensifera insect’s biology.

## Data Availability

DNA-seq data have been deposited in the National Genomics Data Center repository (Bioproject ID: PRJCA013546; BioSample accession: SAMC1008585).

## Supplementary Materials

The following supporting information can be downloaded at: https://www.mdpi.com/article/10.3390/genes14020255/s1, Supplementary Materials File S1: Frequency distribution peaks in the histograms of k-mer abundances in G. orientalis; Figure S1: The k-mer frequency distribution curve of sequencing reads by KmerGenie; Figure S2: GenomeScope result of mole cricket genome; Table S1: Genome size estimation for different values of k-mer using KmerGenie; Table S2: Microsatellites in the Mole cricket.
