# Supplementary figures and images for: Genome Survey Sequencing of the Mole Cricket Gryllotalpa orientalis
Source: Genes (Basel). 2023 Jan 18;14(2):255. doi: 10.3390/genes14020255 (PMC9957284; doi:10.3390/genes14020255)

Number of genomic k-mers

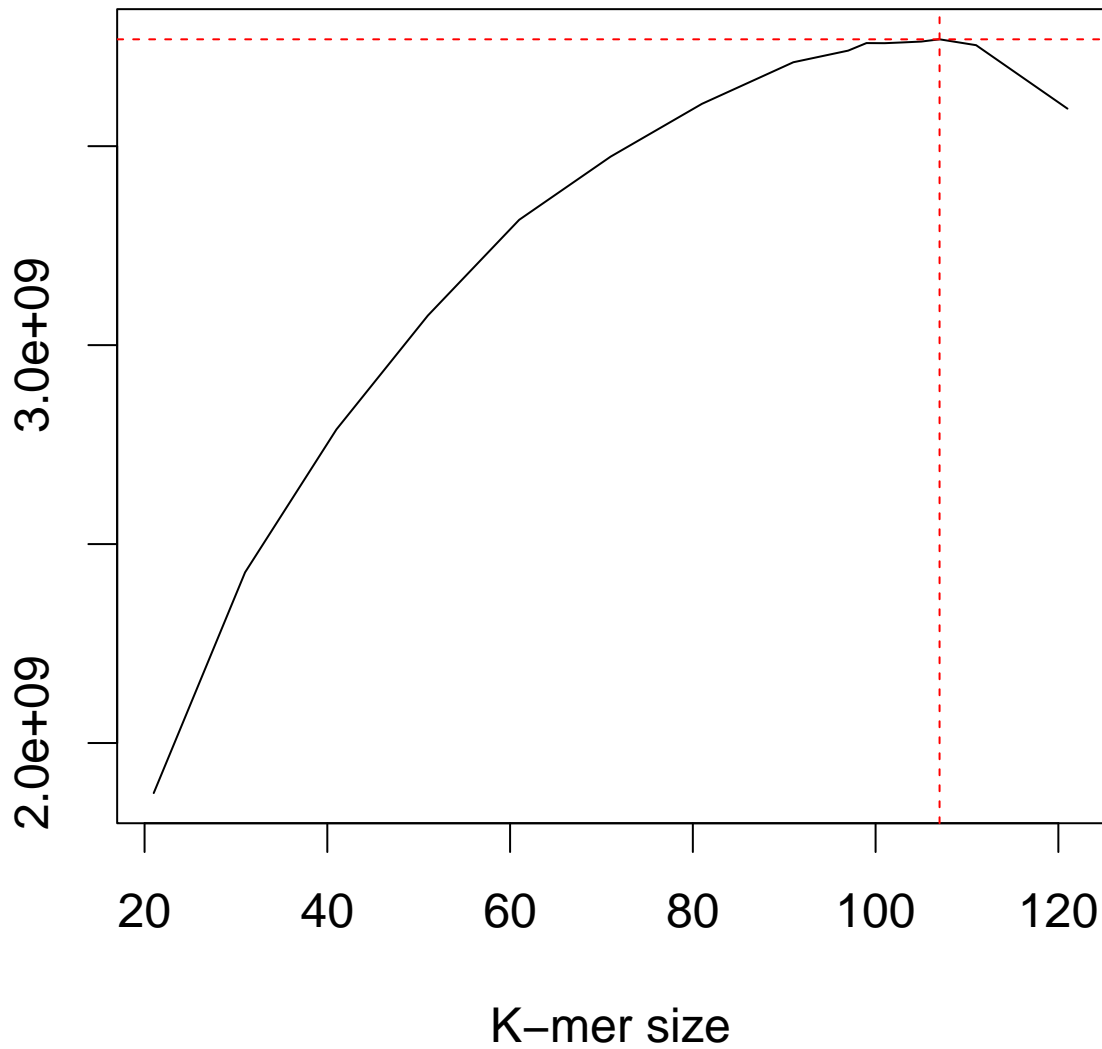

Supplement: Supplementary file 1 [file genes-14-00255-s001.zip › Fig S1.pdf]

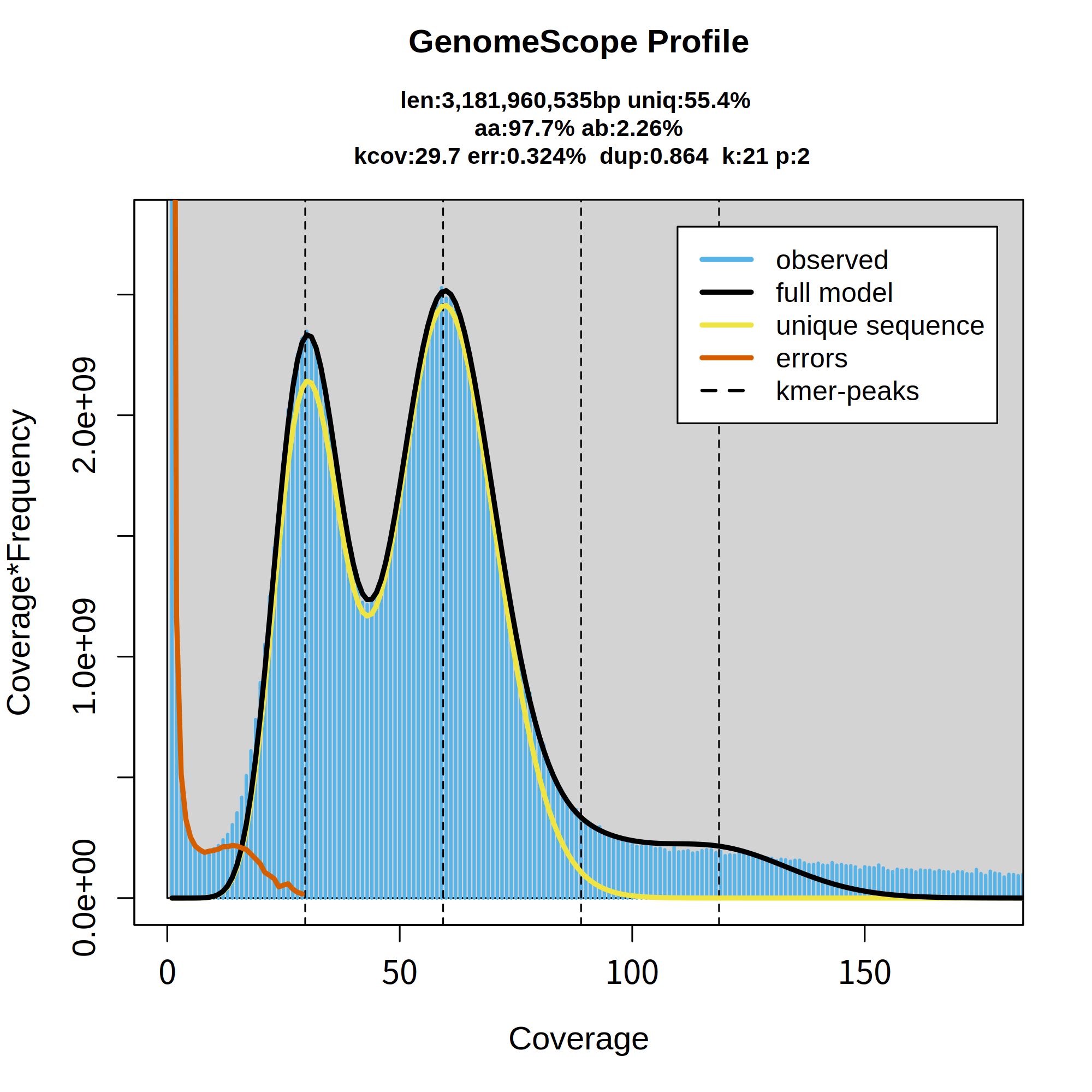

Supplement: Supplementary file 1 [file genes-14-00255-s001.zip › Fig S2.png]

k= 21

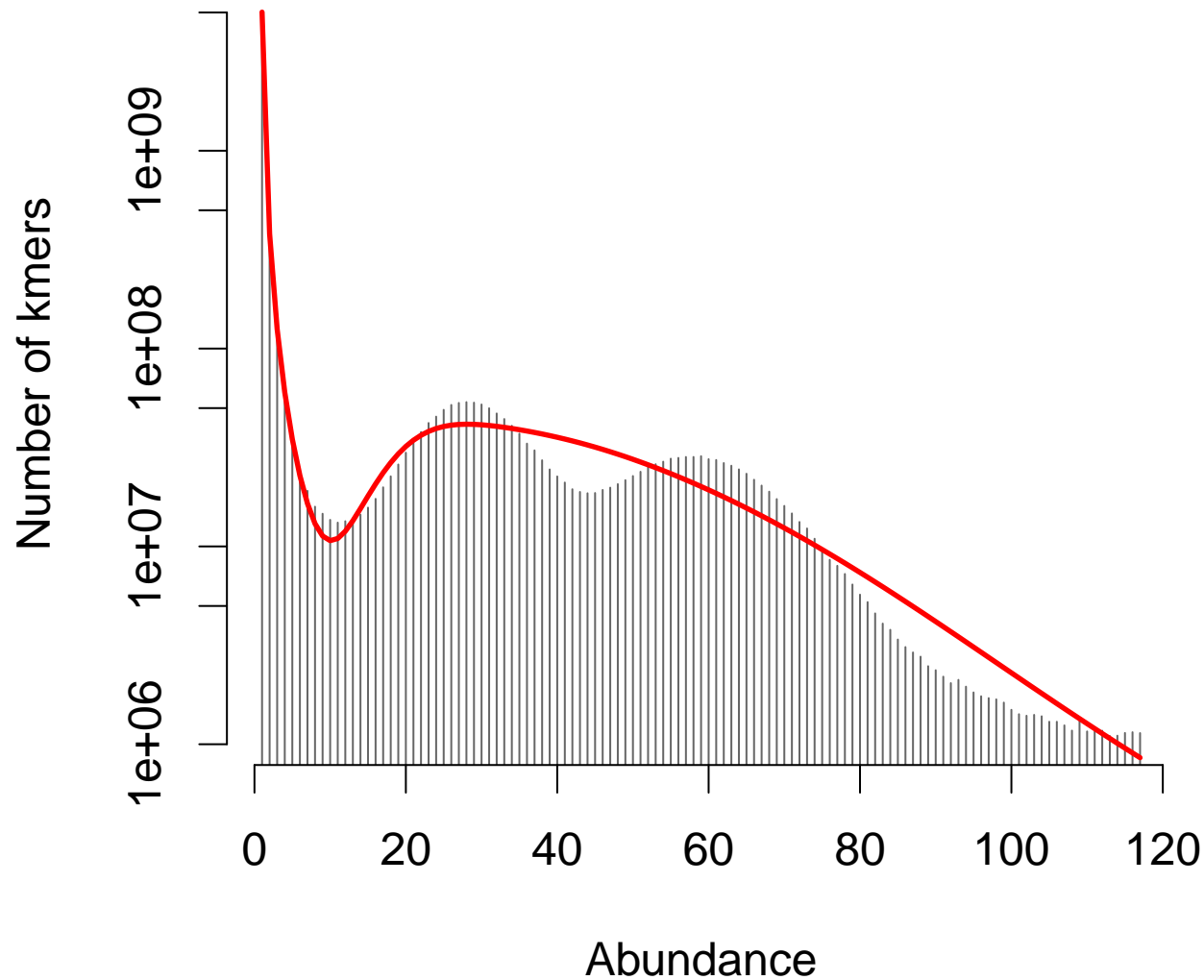

k= 31

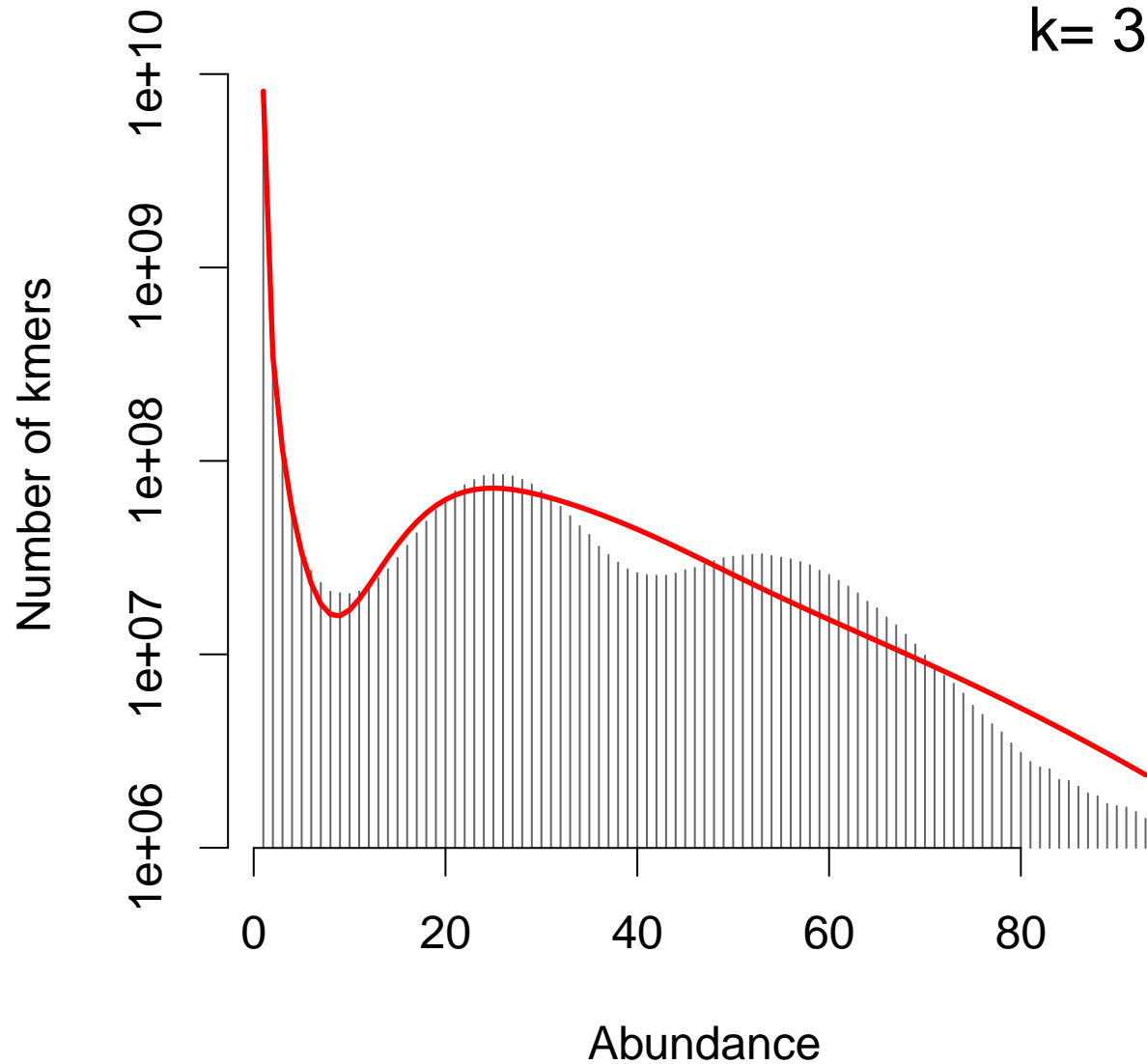

k= 41

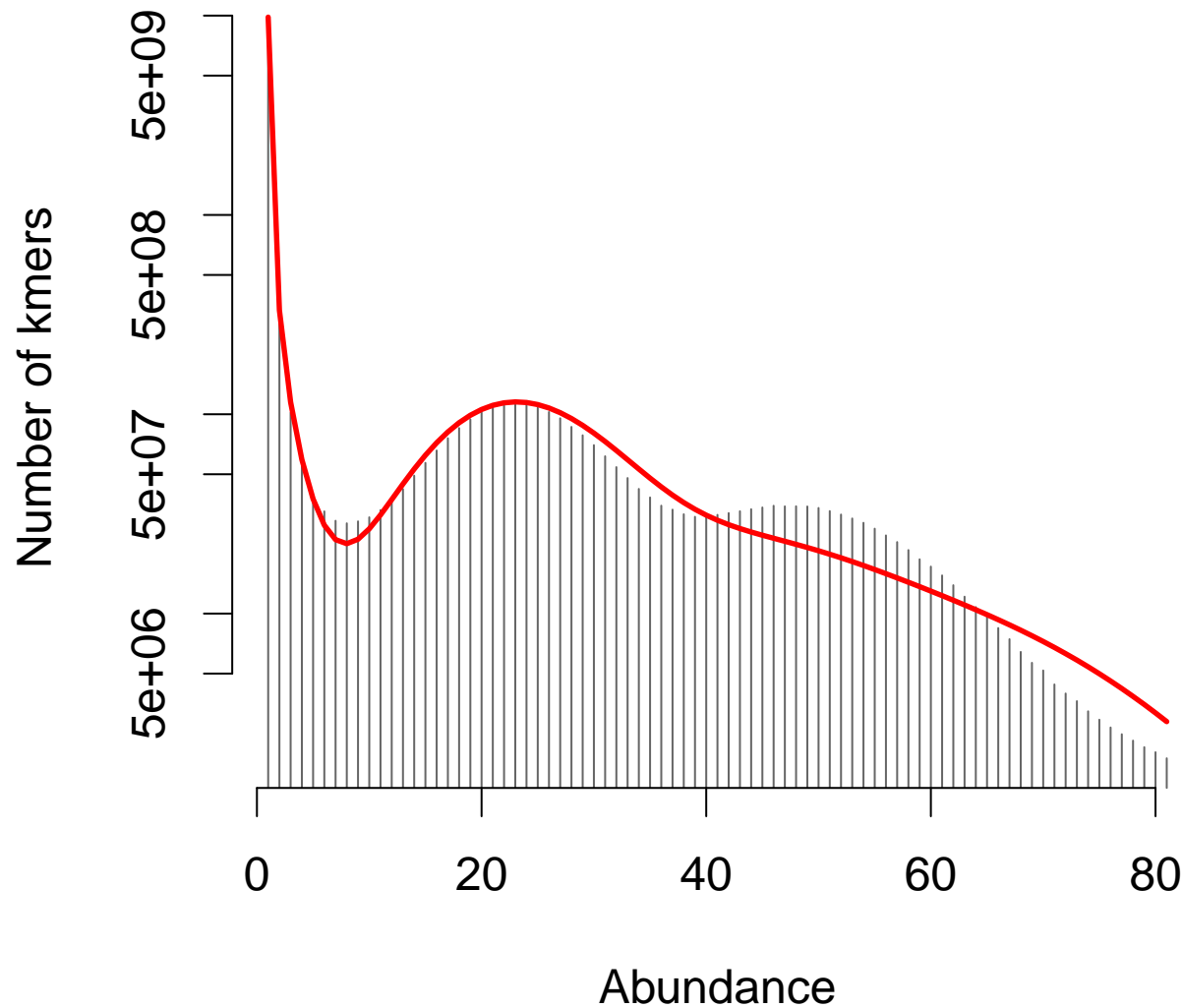

k= 51

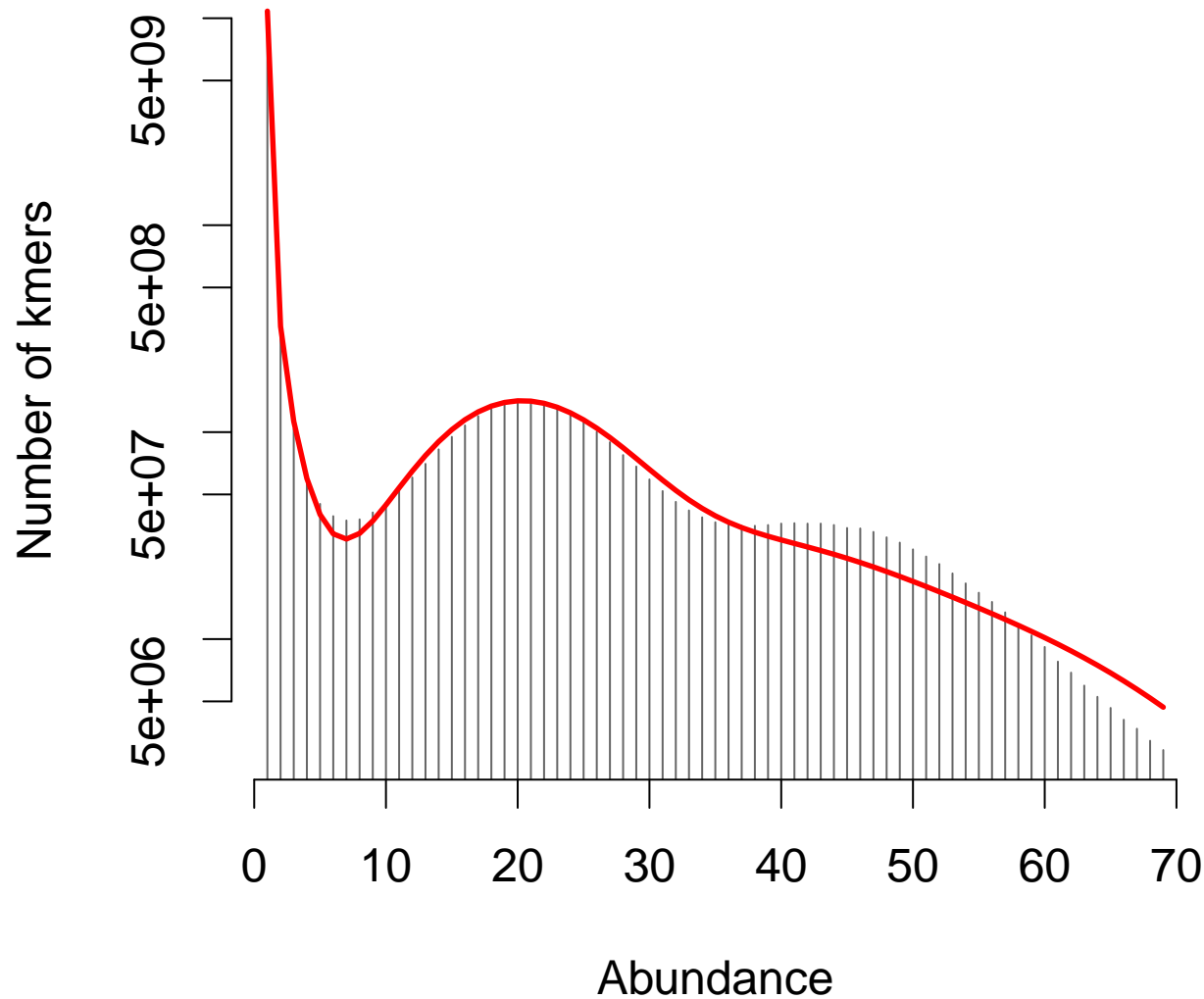

k= 61

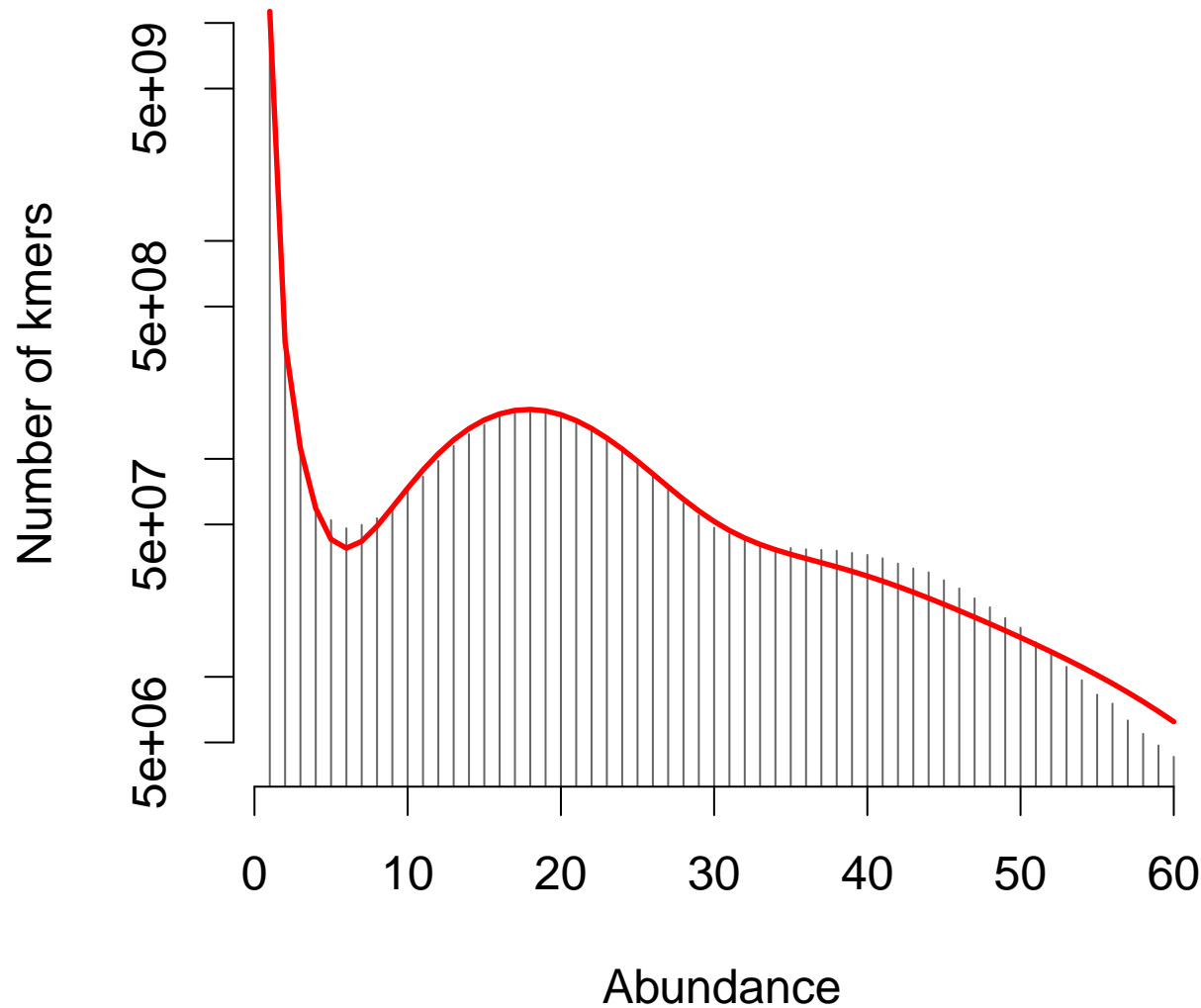

k= 71

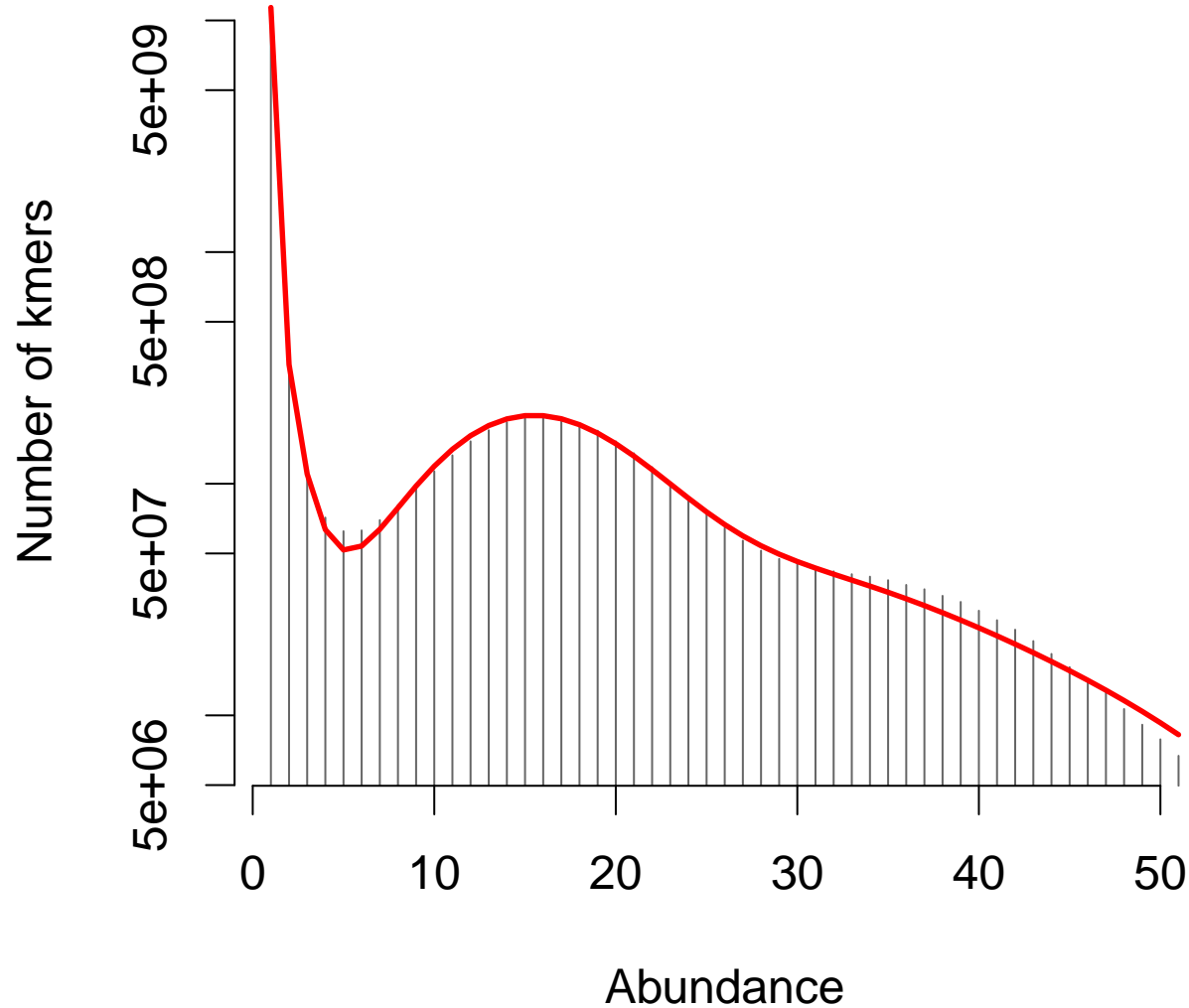

k= 81

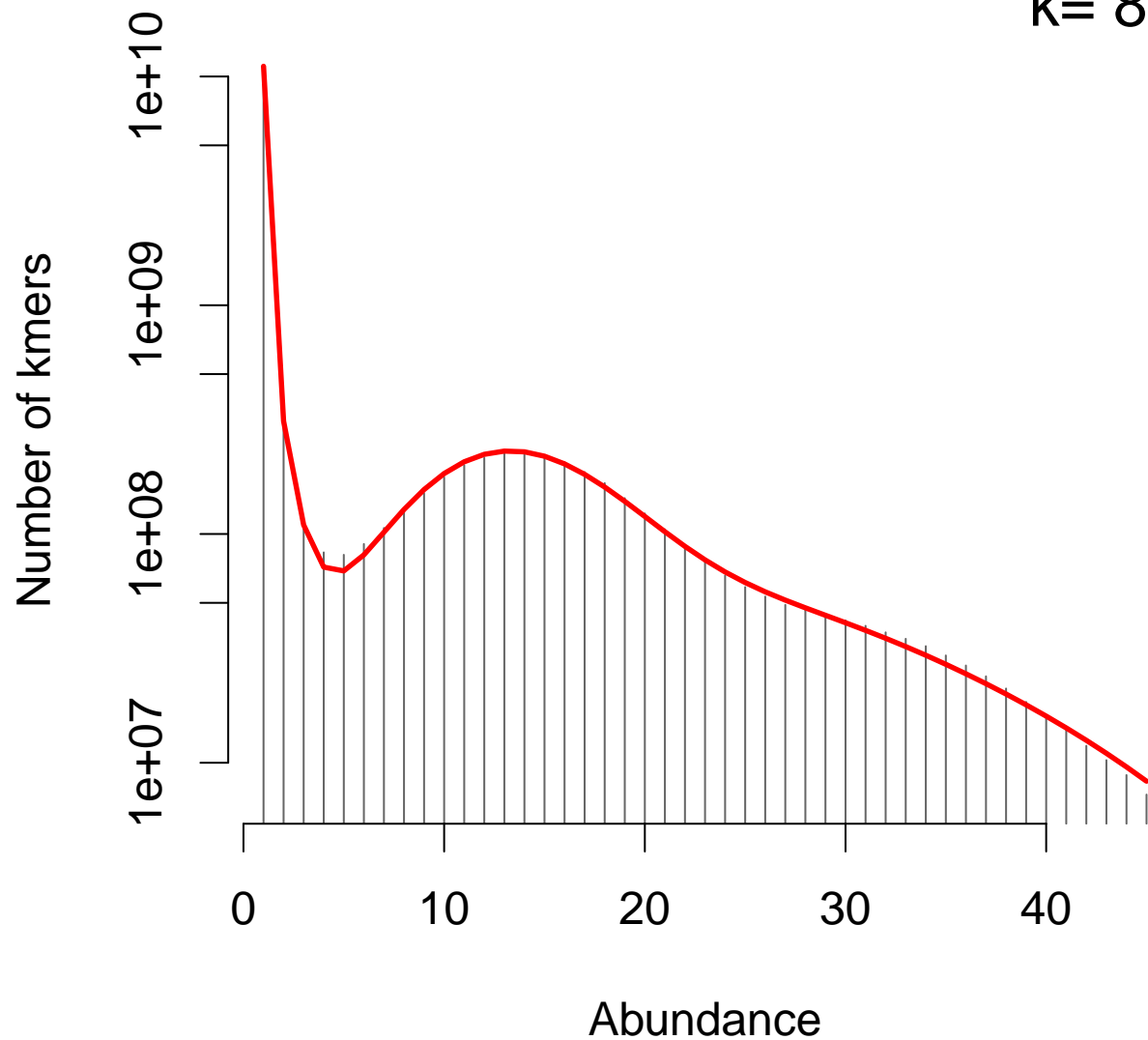

k= 91

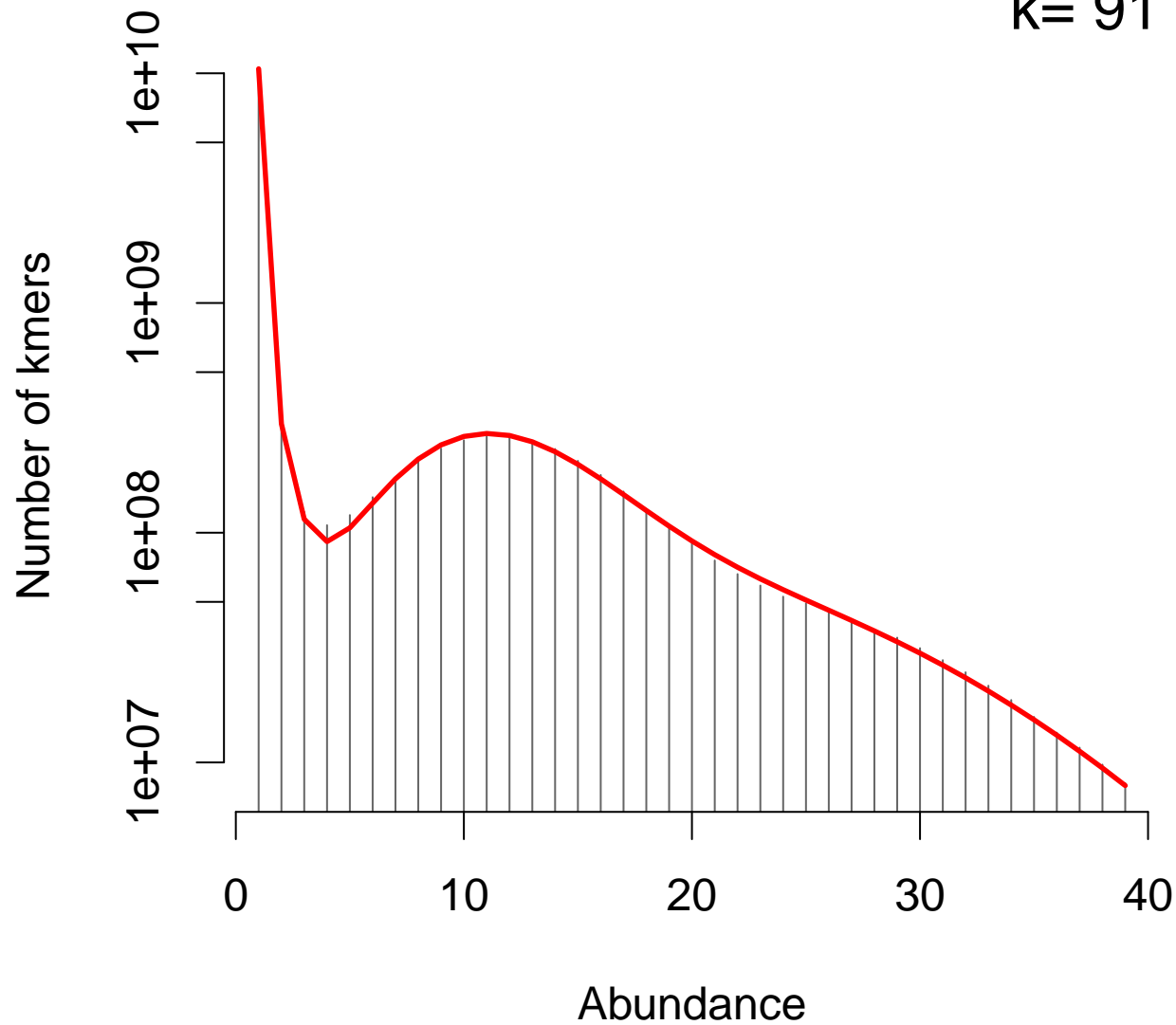

k= 97

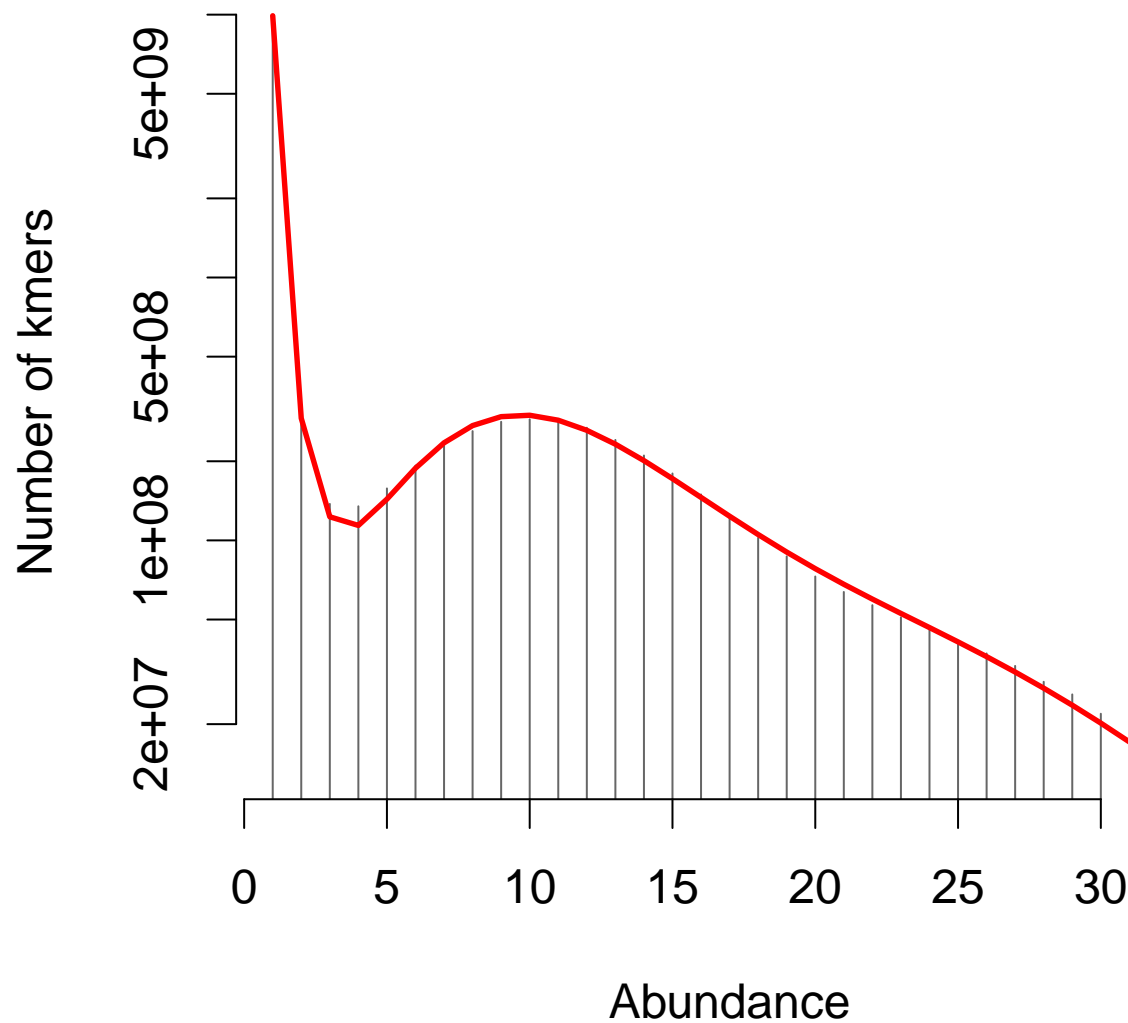

k= 99

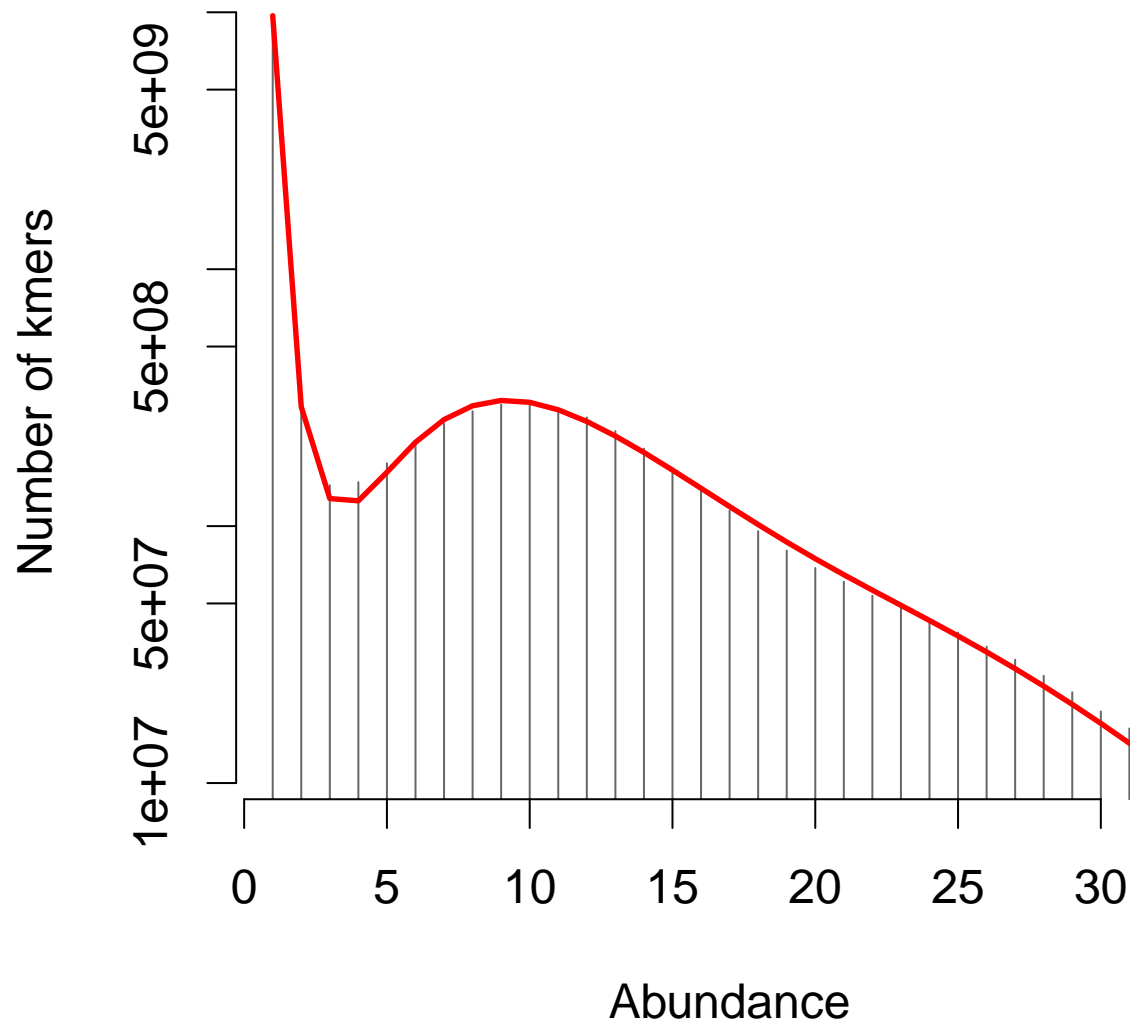

k= 101

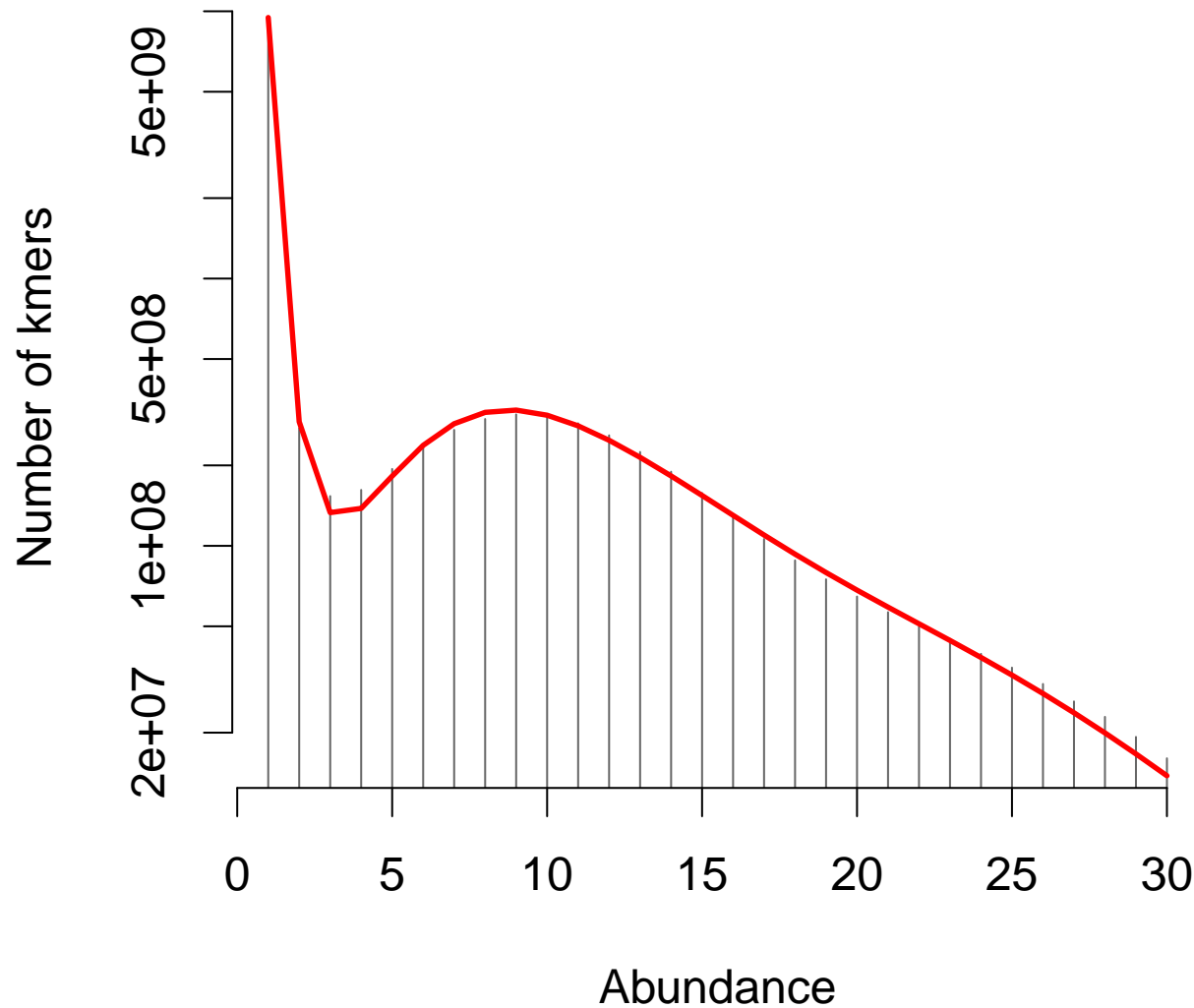

k= 103

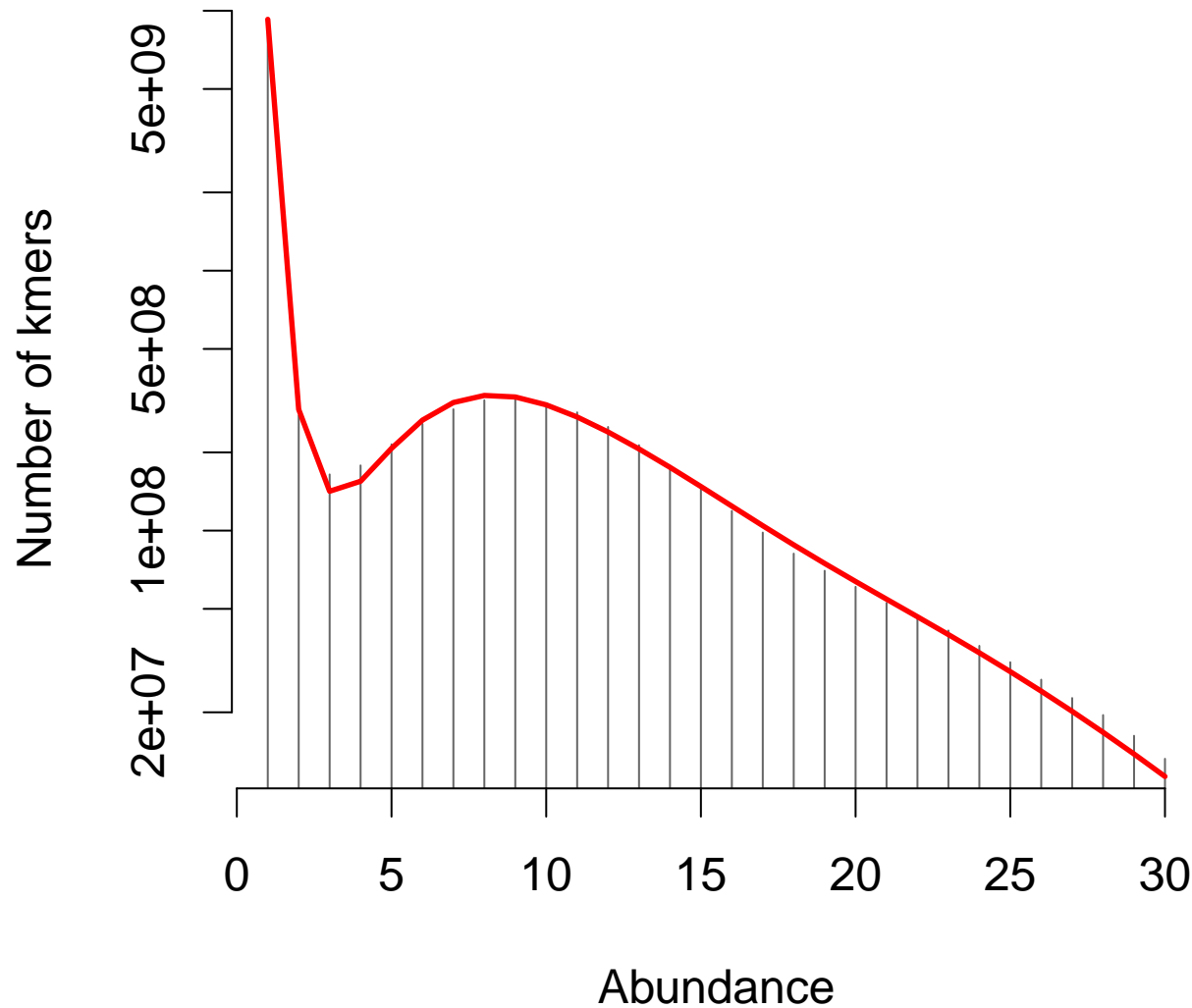

k= 105

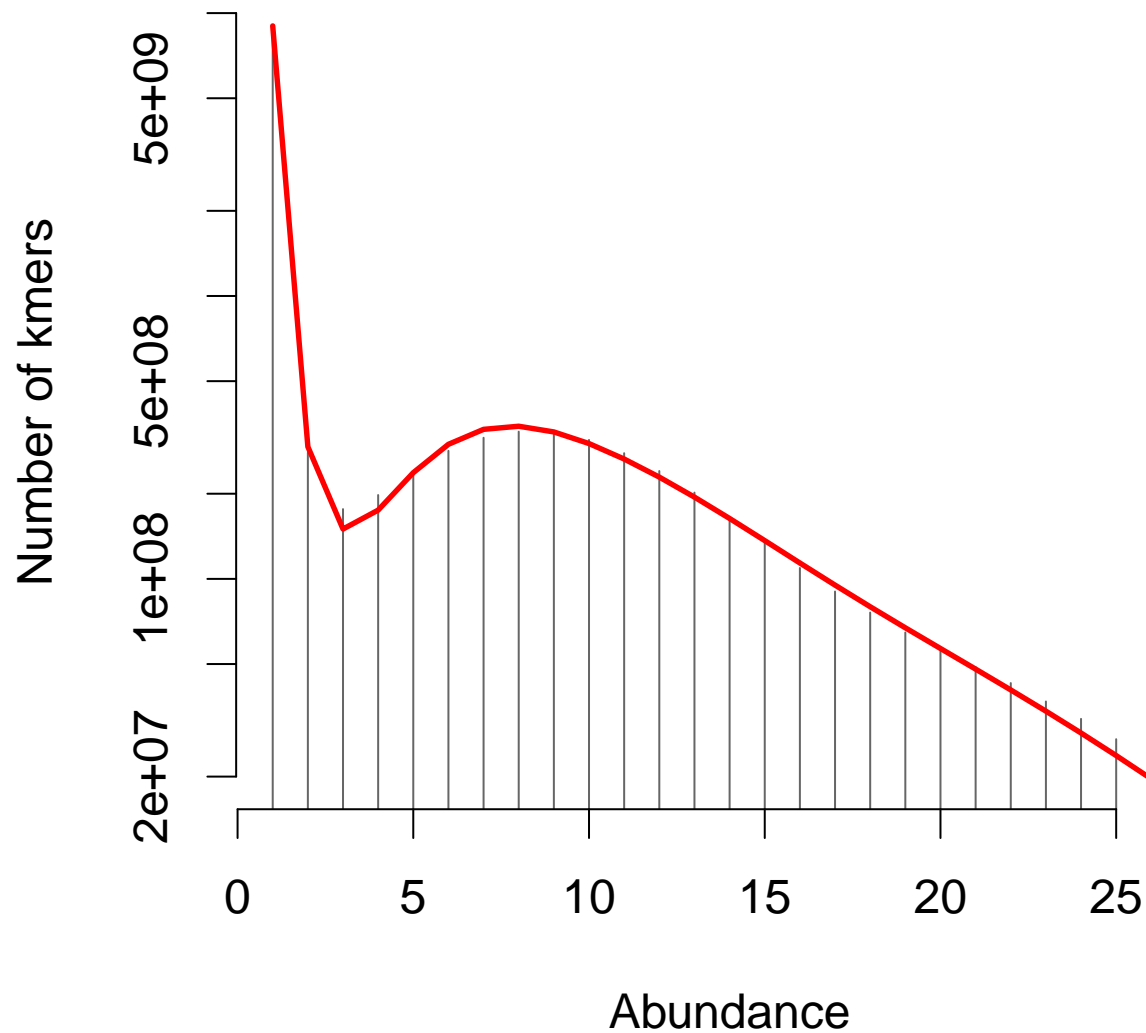

k= 107

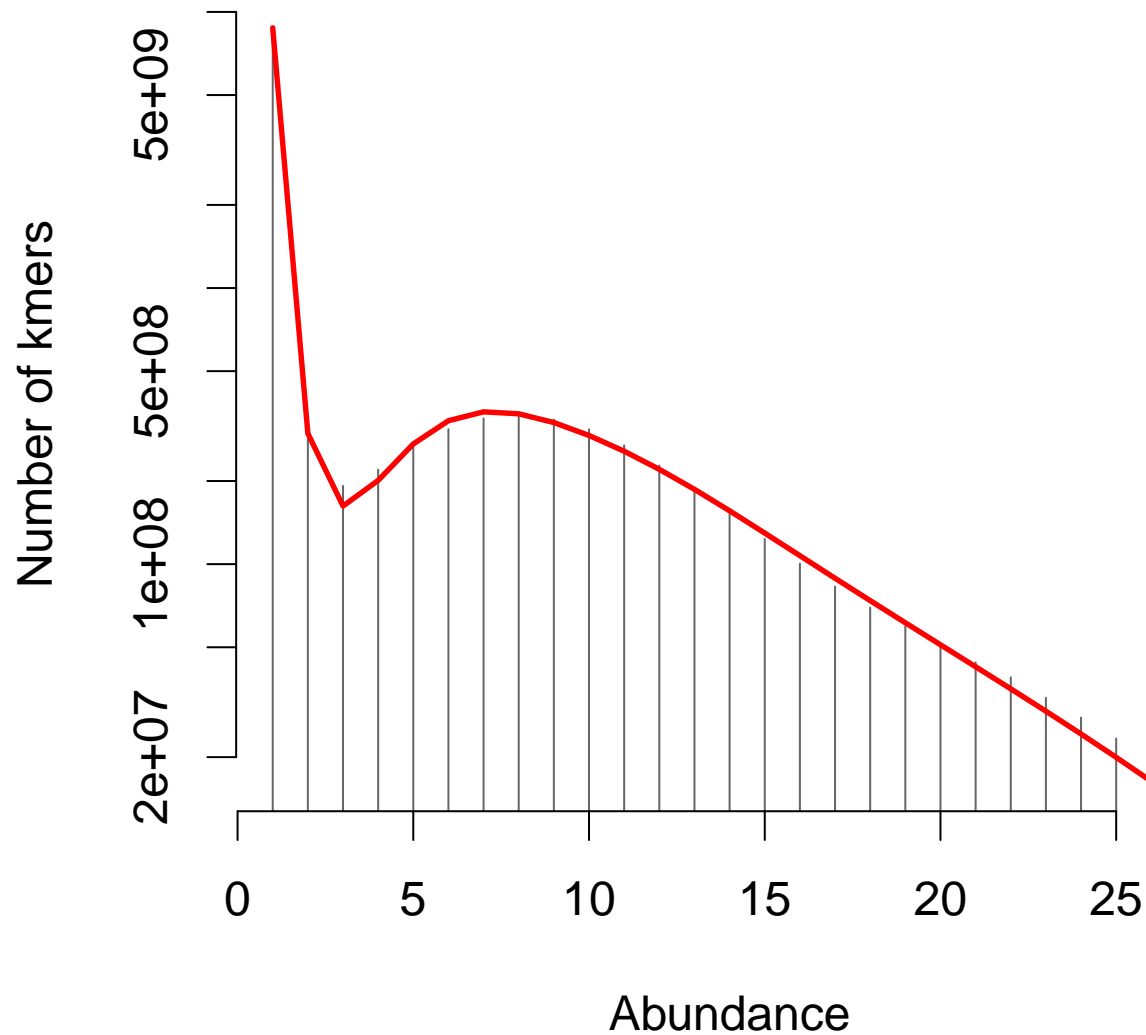

k= 111

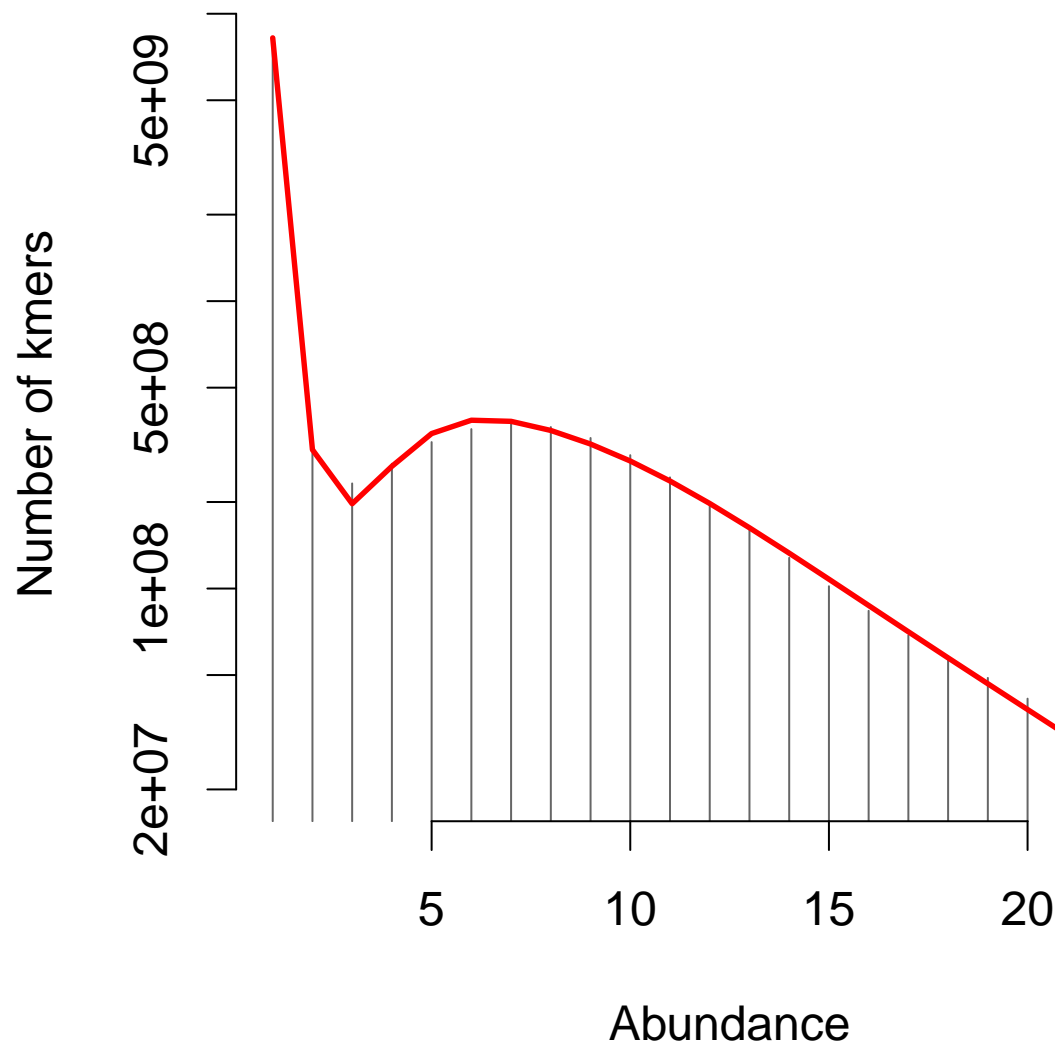

k= 121

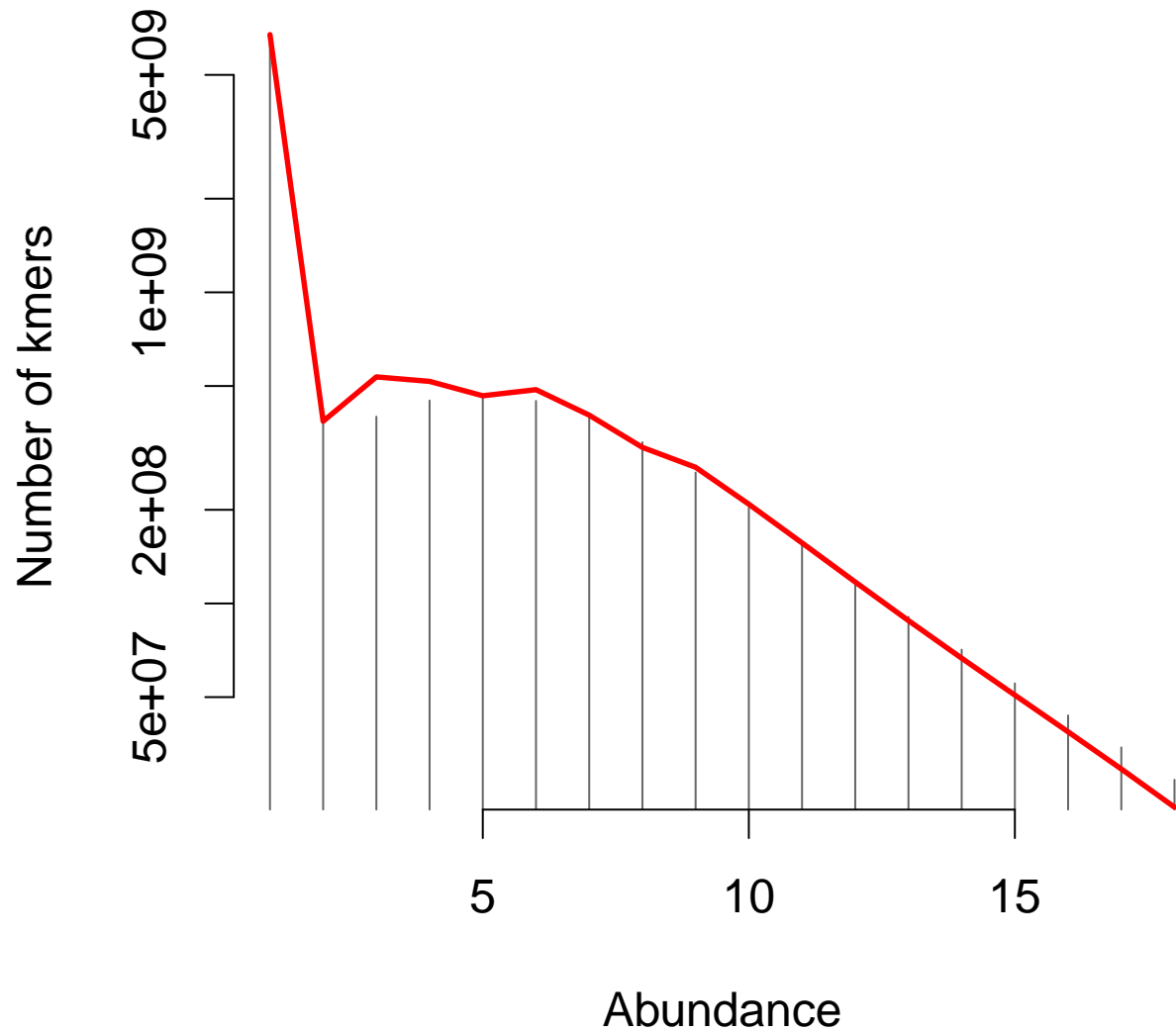

Supplement: Supplementary file 1 [file genes-14-00255-s001.zip › Supplementary Materials File S1.pdf]
